# Empirical model for estimating dengue incidence using temperature, rainfall, and relative humidity: a 19-year retrospective analysis in East Delhi

**DOI:** 10.4178/epih.e2016052

**Published:** 2016-11-27

**Authors:** Vishnampettai G. Ramachandran, Priyamvada Roy, Shukla Das, Narendra Singh Mogha, Ajay Kumar Bansal

**Affiliations:** 1 Department of Microbiology, University College of Medical Sciences and Guru Teg Bahadur Hospital, Delhi, India; 2 Department of Biostatistics and Medical Informatics, University College of Medical Sciences and Guru Teg Bahadur Hospital, Delhi, India

**Keywords:** Dengue, Empirical model, Rainfall, Humidity, Temperature

## Abstract

**OBJECTIVES:**

*Aedes* mosquitoes are responsible for transmitting the dengue virus. The mosquito lifecycle is known to be influenced by temperature, rainfall, and relative humidity. This retrospective study was planned to investigate whether climatic factors could be used to predict the occurrence of dengue in East Delhi.

**METHODS:**

The number of monthly dengue cases reported over 19 years was obtained from the laboratory records of our institution. Monthly data of rainfall, temperature, and humidity collected from a local weather station were correlated with the number of monthly reported dengue cases. One-way analysis of variance was used to analyse whether the climatic parameters differed significantly among seasons. Four models were developed using negative binomial generalized linear model analysis. Monthly rainfall, temperature, humidity, were used as independent variables, and the number of dengue cases reported monthly was used as the dependent variable. The first model considered data from the same month, while the other three models involved incorporating data with a lag phase of 1, 2, and 3 months, respectively.

**RESULTS:**

The greatest number of cases was reported during the post-monsoon period each year. Temperature, rainfall, and humidity varied significantly across the pre-monsoon, monsoon, and post-monsoon periods. The best correlation between these three climatic factors and dengue occurrence was at a time lag of 2 months.

**CONCLUSIONS:**

This study found that temperature, rainfall, and relative humidity significantly affected dengue occurrence in East Delhi. This weather-based dengue empirical model can forecast potential outbreaks 2-month in advance, providing an early warning system for intensifying dengue control measures.

## INTRODUCTION

The dengue virus (DENV) is an arbovirus belonging to the *Flaviviridae* family, and is a cause of classical dengue fever (DF), dengue haemorrhagic fever (DHF), and dengue shock syndrome (DSS), which are major public health problems in Delhi, India [1-3]. These clinical entities are caused by four DENV serotypes (DENV-1, DENV-2, DENV-3, and DENV-4), transmitted to humans by female *Aedes* mosquitoes (*Aedes aegypti* and *Aedes albopictus*) [3,4]. According to the 2014 to 2015 report of the National Vector Borne Disease Control Programme, dengue is endemic in 35 states and Union territories, including Delhi [5]. Dengue is being increasingly reported in urban areas, mainly due to deficient water management, including improper water storage practices, and inconsistent attention to the elimination of vector breeding sites [5].

All four serotypes of DENV circulate in Delhi [6]. Until 2003, the predominant serotype in Delhi was DENV-2, but in 2003, Delhi became a hyperendemic state with all four DENV serotypes co-circulating. During the 2004 epidemic of DF, an abrupt shift occurred, leading to the dominance of DENV-3 at the expense of the previously circulating serotype DENV-2, followed by the complete preponderance of DENV-3 in 2005 and 2006 [6,7]. Over the next four years (2007 to 2010), DENV-1 emerged as the principal serotype [6,7]. DENV-2 was the preponderant serotype from 2011 to 2014 [8]. In 2015 all four serotypes were found to co-circulate, with DENV-2 predominant [9].

The *Aedes* mosquito is a climate-sensitive vector that predominantly affects tropical countries due to their climatic conditions [1,2]. Dengue cases are influenced by complex interactions of humans, vectors, the environment, and virus-related factors [10]. Studies have reported a strong and consistent relationship between the climate of a particular geographical area and the number of dengue cases [11]. Different models have been developed to predict dengue outbreaks by correlating dengue cases with climatic data [12,13]. Amongst the different climatic parameters, rainfall, temperature, and humidity have been reported to be the most important factors influencing DENV transmission. Moreover, in geographical regions where minimum thresholds of these climatic variables are adequate to sustain DENV transmission, seasonal fluctuations in these parameters act as essential determinants of the strength and period of transmission [14]. In recent years, the accuracy of predicting local weather and epidemics has improved due to advances in technology [10]. This has helped gain an understanding of the interaction between climate and the temporal-spatial distribution of infectious diseases, as well as encouraging research interest on epidemic prediction modelling [10,15]. Although dengue prediction models have been developed in many countries across the world, no such study has been reported in India [1, 12,13]. Thus, the present study was planned to develop an empirical model to predict monthly dengue cases using 19 years’ monthly data on the number of dengue cases and three major climatic factors (rainfall, temperature, and humidity) at an 1,800-bed tertiary care hospital in East Delhi.

## MATERIALS AND METHODS

In the present retrospective study, the number of monthly dengue cases reported at Guru Teg Bahadur Hospital, an 1,800-bed tertiary-care hospital in East Delhi, for a period of 19 years (from January 1997 to December 2015) was obtained. Guru Teg Bahadur Hospital is the largest hospital of the government of the National Capital Territory of Delhi in the Trans-Yamuna Area (East Delhi), with a capacity of 1,800 beds. Of the population of Delhi of 12 million people, 12.07% live in East Delhi. Guru Teg Bahadur Hospital is the only Delhi Government tertiary care hospital in the Trans-Yamuna (East Delhi) area, catering to the population of East Delhi as well as patients from the adjacent districts of Noida, Meerut, Loni, Baghpat, and Bulandshahar. Hence, this hospital handles the majority of the dengue cases in East Delhi, as is reflected in the data from 2015, when Guru Teg Bahadur Hospital encountered 1,633 of the 1,737 dengue cases reported in East Delhi in 2015.

This hospital accepts all cases of suspected DF irrespective of severity. In this study, dengue cases were defined and classified according to the National Guidelines for Clinical Management for DF released by the Government of India in December 2014 [16]. The guidelines classify dengue into undifferentiated DF and severe DF based on clinical manifestations. Non-severe dengue cases include DF and DHF grades I and II, while severe dengue includes DHF grades III and IV and DSS. The clinical criteria for DF, DHF, and DSS given in the guidelines are as follows:

### Clinical features of dengue fever

An acute febrile illness of two to seven days’ duration with two or more of the following manifestations: headache, retro-orbital pain, myalgia, arthralgia, rash, and haemorrhagic manifestations.

### Dengue haemorrhagic fever

A case with the clinical criteria of DF plus haemorrhagic tendencies (evidenced by: positive tourniquet test or petechiae, ecchymoses, or purpura, or bleeding from mucosa, gastrointestinal tract, injection sites, or other sites) plus thrombocytopenia (<100,000 cells/mm³) plus evidence of plasma leakage due to increased vascular permeability (manifested by a rise in average haematocrit for age and sex of >20% or a drop of more than 20% in haematocrit following volume replacement treatment compared to baseline or signs of plasma leakage (pleural effusion, ascites, hypoproteinaemia [total serum protein level <6 g/dL]) ≤ 20%

### Dengue shock syndrome

All the above criteria for DHF with evidence of circulatory failure manifested by rapid and weak pulse and narrow pulse pressure (mmHg) or hypotension for age, cold and clammy skin, and restlessness.

The protocol of this study was approved by the University College of Medical Sciences Institutional Review Board. Informed consent was obtained from the study subjects. A diagnosis of dengue was made on the basis of clinical findings and serology. Serological confirmation of DF during the study period was carried out using the following tests:

a) Panbio (Brisbane, Australia) Dengue Duo Cassette, a rapid diagnostic test, was used to detect anti-dengue immunoglobulin G (IgG) and immunoglobulin M (IgM) antibodies from January 1997 to December 2001.
b) Panbio (Brisbane, Australia) Dengue IgM capture enzymelinked immunosorbent assay (ELISA) was employed from January 2002 to August 2006.
c) IgM dengue capture ELISA (MAC-ELISA) kits supplied by the National Institute of Virology, Pune under the aegis of the National Vector Borne Disease Control Programme were introduced in September 2006 and have been in use since then.
d) SD Bioline Dengue Duo (non-structural protein 1 [NS1] and IgG/IgM) (Standard Diagnostics Inc., Seoul, Korea) was used from October 6, 2012 to December 31, 2013. The IgM-positive samples were confirmed by MAC-ELISA.
e) NS1 antigen capture ELISA from Panbio (Brisbane, Australia) was introduced on October 4, 2014 and used along with MAC-ELISA for the serological confirmation of DF.

Monthly climatic data (rainfall, temperature, and humidity) was collected from the Delhi Weather Station, Safdarjung, New Delhi (http://www.en.tutiempo.net/climate/ws-421820-html). The monthly climate data were correlated with the number of monthly reported dengue cases.

Average monthly rainfall, temperature, and humidity were used as independent variables and the number of dengue cases reported monthly was used as the dependent variable. One-way analysis of variance (ANOVA) was used to determine whether each of the climate variables differed significantly between seasons.

An empirical model was developed using negative binomial generalized linear model analysis. A negative binomial model with a log-link function in the generalized linear model was used to obtain the models for estimating dengue cases based on the independent variables of rainfall, temperature, and humidity. Since a significant correlation was present in the variability of dengue cases across seasons, we included season as a covariate and obtained modified models. A negative binomial model was used because our outcome variable was measured as counts and was over-dispersed; that is, the conditional variance was quite high compared to the conditional mean.

When several maximum likelihood models are available, one can compare the performance of alternative models based on several likelihood or goodness of fit measures. Two of the most regularly used measures are the Akaike information criterion (AIC) and the likelihood ratio chi-square. The model exhibiting maximum change in the value of AIC and likelihood ratio chi-square compared to the previous model was considered to be the best-fitting model.

## RESULTS

**Figure 1.**
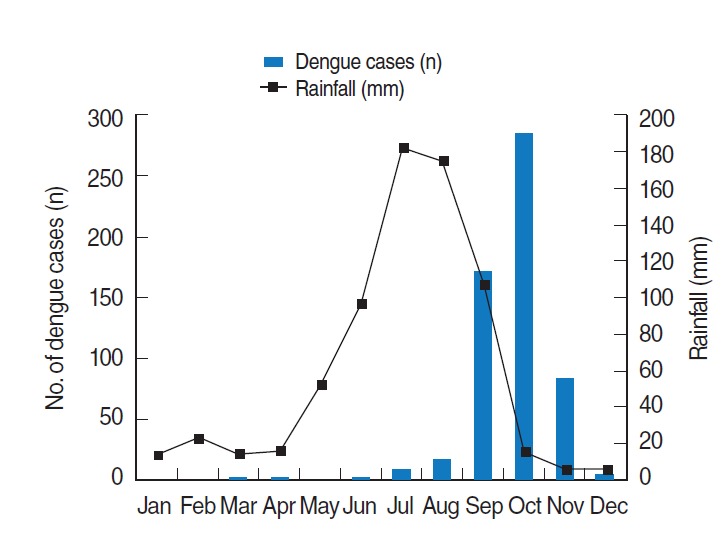
Average number of dengue cases and rainfall by month from 1997 to 2015.

**Figure 2.**
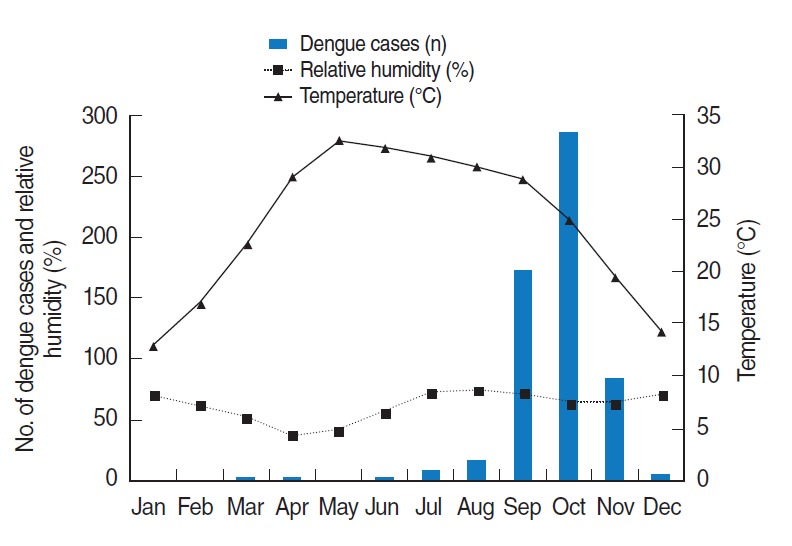
Average number of dengue cases, temperature, and relative humidity by month from 1997 to 2015.

Over the 19-year period (1997 to 2015), a total of 6,703 inpatient and outpatient cases of DF, including severe forms of the disease such as DHF and DSS, were reported at Guru Teg Bahadur Hospital. The number of reported dengue cases varied by year (Table 1). Over the study period, the highest number of dengue cases was reported in 2015 (n=1,633). Every year, the occurrence of dengue cases displayed a particular pattern. During the pre-monsoon season, hardly any cases of dengue were reported. Most of the cases were reported during the post-monsoon period each year, except in 2010, when the highest number of cases was reported during the monsoon. The average number of dengue cases per month (January to December) over the 19-year period (1997 to 2015) was plotted against the climatic factors (rain, temperature, and relative humidity) to assess their influence on the occurrence of DF (Figures 1 and 2). Every year, a bellwether of cases was reported in the month of July, reaching a peak in September and October, and gradually declining at the end of the year (Table 1).

Temperature, rainfall, and humidity varied significantly over the pre-monsoon, monsoon, and post-monsoon periods every year (Table 2). The average monthly pre-monsoon, monsoon, and post-monsoon rainfall amounts were 28.72 mm, 150.69 mm, and 11.02 mm, respectively, over the 19-year period (1997 to 2015). The average temperature recorded during the pre-monsoon, monsoon, and post-monsoon period was 25.53˚C, 30.80˚C, and 18.20˚C, respectively. The average relative humidity recorded during the pre-monsoon, monsoon, and post-monsoon period was 49.87%, 68.68%, and 68.83%, respectively. The overall variation in all three climatic variables was statistically significant (ANOVA, p<0.001). On applying cross-correlation analysis between the three climatic factors and dengue occurrence, the best correlation was found at a time lag of 2-month for all three variables.

### Development of an empirical model

The number of dengue cases increased in the post-monsoon period, indicating a correlation between dengue infection and climatic factors (rainfall, temperature, and relative humidity), as well as providing a basis for a possible empirical model of dengue. Four models were developed using a negative binomial generalized linear model (Table 3). The first model was developed considering data from the same month. Under the influence of climatic factors, it takes 7 to 45 days for an adult mosquito to develop from an egg [8]. Hence, the influence of climatic factors was expected to manifest with a lag of 1-2 months. Therefore, three other models were generated, incorporating a lag phase of 1, 2, and 3 months, respectively. It was observed that the AIC decreased and the likelihood ratio chi-square increased from model 1 to model 2, and then from model 2 to model 3. However, the difference in both parameters was not significant between model 3 and model 4. Thus, model 3 was chosenas the final model.

Model 3, 2-month time lag: log D_m_=rainfall_m_×0.001+temperature_m_×0.501+humidity_m_×0.152-22.037; AIC=856.708, likelihood ratio chi-square=849.017.

Another set of models was prepared including season as a covariate in addition to rainfall, temperature, and relative humidity (Table 4). It was found that the AIC decreased and the likelihood ratio chi-square increased from model 1 to model 2, but the difference in the two parameters was minimal between model 2 and model 3. Hence, model 2 could be chosen as the final model if season was also considered as a covariate.

Model 2, 1-month time lag: log D_m_=Rainfall_m_×0.012+temperature_m_×0.314+humidity_m_×0.058+season×4.794-23.025; AIC =1,020.722, likelihood ratio chi-square=956.592.

## DISCUSSION

The growth and development of dengue vectors depends on weather conditions. As dengue is a vector-borne disease, the occurrence of dengue infections depends on the presence and density of its vector [1]. Numerous studies conducted worldwide have proposed that climatic factors, with temperature, rainfall, and humidity being the most important, are the reason for seasonal variation in the presence of both the vector *Aedes aegypti* and DENV [1, 3,14,17].

In the present study, we found a relationship between dengue cases and rainfall. This is consistent with some other studies [1, 3,17]. Focks & Barrera [17] asserted that vector density increases due to rainfall, causing an increase in dengue cases, which in turn because higher humidity during the rainy season provides an ideal environment for the growth and survival of mosquitoes. However, the few studies in the literature on the association between rainfall and dengue have reported contradictory findings, since the correlation depends on local characteristics [2,18].

This study demonstrated a correlation between the incidence of DF and average temperature. Similar findings were reported by Karim et al. [1] and Chandy et al. [3], while contrasting results were reported in a study by Su [13]. Temperature is a crucial limiting factor in the maturation of the dengue vector. Studies have estimated the threshold survival temperature for the dengue virus to be 11.9°C and have demonstrated that *Aedes aegypti* ceases to feed when the temperature falls below 17°C, along with non-amplification of the virus in the vector when the temperature falls below 18°C [2]. Therefore, at low temperatures, the mosquito does not live long enough to become infectious and transmit the virus, and the virus does not develop properly. Viral replication and the extrinsic period in the insects are shortened by high temperature. A reduced viral incubation time increases the probability of the vector living long enough to transmit the virus, thereby magnifying epidemics. Hence, temperature analysis improves our understanding of dengue epidemiology [2]. Increases in global temperatures may expand the area of involvement and number of cases of vector-borne diseases [1,13], and will increase the proportion of infective mosquitoes, giving rise to an exponential increase in dengue cases [1]. It has also been shown that a temperature increase from 26˚C-28˚C to 30˚C decreases the extrinsic incubation of the dengue virus, which may promote viral transmission [1,19,20].

The present study demonstrated an association between relative humidity and dengue cases. Relative humidity is influenced by a combination of rainfall and temperature. This important weather parameter affects the lifespan of mosquitoes and hence, viral transmission. Karim et al. [1] and Promprou et al. [21] found more dengue cases during the monsoon when the relative humidity is higher. Higher humidity during the rainy season encourages the development and propagation of mosquitoes, causing an increase in the number of infected mosquitoes [1,22]. Barbazan et al. [22] have postulated that an increase in the mosquito lifespan disproportionately augments the frequency of potential transmissions by as much as five times when the survival rate rises from 0.80 to 0.95. Hales et al. [23] have also reported that the annual average vapour pressure was the most important climatic predictor of global dengue occurrence.

The cumulative effect of temperature and humidity strongly influences the number of mosquito blood meals, survival rate of the vector, and the likelihood of a mosquito becoming infected with DENV [14]. According to studies conducted worldwide, relative humidity and temperature are the most important climatic predictors of changes in dengue transmission [14,24,25]. Hence, through their effects on the *Aedes* vector, rainfall, temperature, and relative humidity are essential factors determining the geographic areas within which dengue transmission can be expected to occur [14].

In the present study, significant differences were found amongst the pre-monsoon, monsoon, and post-monsoon periods in the amount of rainfall, relative humidity, and average temperature (Table 2). Dengue cases peaked during the post-monsoon period, with a time lag of 1 and 2-month in models developed including and excluding season as a covariate, respectively (Tables 3 and 4). This could be accounted for by the indirect effect of climatic factors on the incidence of dengue through their influence on the lifecycle of both the vector and virus [14]. Climatic factors affect mosquito hatching, larval and pupal development, and the emergence of adult mosquitoes, as well as virus amplification and incubation in humans, finally culminating in a dengue outbreak after a cumulative time lag [14,26]. The time lag can be explained by the influence of weather conditions on the biological development of the mosquito vector, including prolonged egg hatching periods and the propensity of *Aedes* eggs to survive without water for many months [10]. Depending on the time lag between the biological cycle and the clinical symptoms, a lag between climate data and dengue incidence data will emerge [14]. Our results agree with those of other studies from diverse geographical regions [11,21,27]. A season-specific pattern of dengue cases in Southeast Asia has been reported [1,28]. A similar lag phase of 2-month for explaining the occurrence of dengue cases was reported by Karim et al. [1] in Dhaka. Chen et al. [29] reported a significant positive correlation of dengue cases with temperature and relative humidity at a lag of 1-3 months in Thailand.

In this study, the greatest number of dengue cases was reported in 2015 (n=1,633). As relative humidity and temperature have been reported to be the most important climatic predictors of changes in dengue transmission, the most ideal combination of high relative humidity and high temperature was present in 2015 in comparison to all other years in the study period, thereby resulting in the greatest number of dengue cases. Several studies have reported that the climate variable indices of the Indian Ocean dipole and El Niño southern oscillation play an important role in the interannual variation in dengue transmission [30,31]. Variations in oceanic sea surface temperature have also been found to be responsible for interannual variation in dengue cases [32]. An enhanced case burden is also attributable to a general apathy to civic hygiene, unmonitored drain cleaning and silting, stagnation of rain water, highly unstructured and ill-supervised mosquito control measures, an excess focus on fogging-based vector control, and the institution of remedial measures only after the problem of an epidemic surfaces. These are supplemented by an excessive migrant population, an inadequate health-care delivery system in the districts adjacent to Delhi, and the excess congregation of ill patients accompanied by healthy caregivers in healthcare facilities, providing a milieu for vector-susceptible contact.

Studies have reported slight indications of a correlation between mosquito status and dengue spread that can be employed to predict dengue outbreaks [33]. We collected Breteau index (BI) data from 2007, 2008, and 2009 from the Municipal Corporation of Delhi (Table 5). The mean BI was very low in the pre-monsoon season. The mean BI reached its maximum levels from September to November, which is consistent with our finding of the peak number of dengue cases in September and October. The mean BI was highest in 2008 among the 3-year, which also correlates with the fact that out of those three years, the greatest number of dengue cases was reported in 2008.

Since this study suggests that temperature, rainfall, and humidity are significantly associated with DF incidence at a time lag of 2-month, mosquito control and dengue surveillance must be strengthened during the post-monsoon season. This window of opportunity provides enough time to mobilize resources for the implementation of interventional measures to minimize the impact of the epidemic [34]. It would permit vector control units to carry out their operations during the high-risk season, thus maximizing limited vector control resources, as well as offering local authorities sufficient time to alleviate a probable outbreak successfully. It also allows the avoidance of vector control measures triggered by false alarms. Disease surveillance measures enabling public health practitioners to be aware of the scale of dengue morbidity and control of mosquitoes are necessary to curtail dengue transmission. The creation of diagnostic centres is essential for facilitating surveillance and providing an early warning of the changes in dengue incidence. Dengue control requires the implementation of an integrated approach, incorporating environmental management, chemical control, and biological methods for the control of mosquitoes [13].

This study has some limitations. Only monthly data were available to us. A weekly analysis of incidence data and weather conditions would help explore the true impact of climatic variability on dengue incidence. Wind velocity is also known to have a positive effect on dengue incidence. However, such data were not available. Another limitation is the use of records from a single hospital as a surrogate for surveillance in a specific area, rather than using direct surveillance data.

This study found that climatic parameters can serve as important components for generating an uncomplicated, accurate, cost-effective, and timely dengue forecasting system. The development of a weather-based dengue forecasting model could assist local vector control, prevention, and surveillance in several ways. The model would act as an early warning system for enhancing measures of dengue control to reduce the size of an outbreak, thereby decreasing disease transmission and possibly the resulting mortality, leading to reductions in the healthcare burden and operating costs. Nonetheless, the long-term sustainability of forecast accuracy remains a potential challenge of the dengue forecasting model, since it assumes that a particular distribution pattern will be repeated in the future, while changes of dengue epidemiology in the long run are unavoidable as factors affecting dengue epidemiology evolve over time. Hence the model may have to be re-calibrated in the future to maintain long-term forecast precision by anticipating alterations in factors influencing dengue transmission and distribution patterns.

## Figures and Tables

**Table 1.** Seasonal variation in the number of dengue cases each year

Year	Pre-monsoon	Monsoon	Post-monsoon	Total
1997	1	1	14	16
1998	0	0	15	15
1999	0	1	4	5
2000	0	13	34	47
2001	0	5	16	21
2002	0	0	0	0
2003	0	0	571	571
2004	0	5	42	47
2005	0	0	14	14
2006	0	58	621	679
2007	0	0	137	137
2008	0	240	304	544
2009	0	14	277	291
2010	0	603	327	930
2011	0	2	11	13
2012	0	45	159	204
2013	0	513	914	1,427
2014	1	3	106	109
2015	0	769	864	1,633

Pre-monsoon, February, March, April, and May; Monsoon, June, July, August, and September; Post-monsoon, October, November, December, and January.

**Table 2.** Distribution of climatic parameters by season (1997-2015)

Year	Mean rainfall (mm)			Mean temperature (°C)			Mean relative humidity (%)		
	Pre-monsoon	Monsoon	Post-monsoon	Pre-monsoon	Monsoon	Post-monsoon	Pre-monsoon	Monsoon	Post-monsoon
1997	20.69	109.90	18.02	23.83	30.15	16.70	48.35	70.98	76.10
1998	13.13	150.51	13.81	25.03	30.70	17.88	50.18	72.10	70.03
1999	3.76	69.24	13.06	25.70	31.03	18.08	44.70	67.45	68.78
2000 2001	16.84 33.53	124.73 134.63	11.74 7.07	25.40 24.85	30.20 30.48	18.70 18.23	50.25 53.78	71.78 69.73	66.70 68.38
2002	37.33	93.35	7.40	26.20	31.48	18.43	45.05	65.35	69.60
2003	9.95	263.20	17.18	25.38	29.35	17.23	46.63	68.25	69.43
2004	35.07	91.00	26.25	26.43	30.95	18.03	47.30	64.00	70.50
2005	22.73	156.68	1.95	25.35	31.10	18.00	45.58	65.78	61.93
2006	21.25	166.30	1.88	26.68	30.70	18.90	47.35	67.53	63.28
2007	52.43	153.48	0.90	25.43	31.03	17.95	53.83	69.80	62.23
2008	51.68	176.23	1.30	25.03	29.80	18.80	50.50	75.70	67.05
2009	20.83	147.00	6.10	25.95	31.63	18.55	47.33	64.05	65.75
2010	8.93	256.98	8.93	27.73	30.73	18.35	47.08	70.90	74.50
2011	22.75	154.00	0.28	25.28	30.48	18.40	54.00	73.18	69.08
2012	13.60	76.10	5.50	25.33	31.65	17.88	45.10	65.28	68.00
2013	11.08	55.65	52.65	25.70	30.65	18.08	42.35	73.40	74.90
2014	41.80	124.30	26.40	24.28	32.05	18.43	58.95	63.25	70.03
2015	63.60	207.68	9.50	25.50	31.08	19.15	58.93	66.50	71.53
Average	28.72	150.69	11.02	25.53	30.80	18.20	49.87	68.68	68.83

**Table 3.** Models based on a negative binomial generalized linear model relating the monthly number of dengue cases with rainfall, temperature, and relative humidity

	Variables	Estimates	Standard error Wald chi-square	
Model 1	Constant	12.553	1.131	123.255
	Rainfall	0.013	0.001	116.645
	Temperature	0.222	0.022	101.035
	Humidity	0.171	0.013	173.947
Model 2	Constant	17.302	1.242	194.201
	Rainfall	0.007	0.001	31.780
	Temperature	0.373	0.025	229.621
	Humidity	0.166	0.013	152.400
Model 3	Constant	22.037	1.665	175.135
	Rainfall	0.001	0.001	0.274
	Temperature	0.501	0.038	174.895
	Humidity	0.152	0.013	129.259
Model 4	Constant	26.331	2.344	126.172
	Rainfall	0.006	0.001	21.306
	Temperature	0.786	0.062	160.617
	Humidity	0.071	0.011	37.879

Model 1, no time lag: log D_m_=rainfall_m_×0.013+temperature_m_×0.222+humi-ditym×0.171−12.553; AIC=1,579.652; likelihood ratio chi-square=190.243; Model 2, 1-month time lag: log D_m_=rainfall_m_×0.007+temperature_m_×0.373+humiditym×0.166−17.302; AIC=1,260.605; likelihood ratio chi-square= 472.393; Model 3, 2-month time lag: log D_m_=rainfall_m_×0.001+temperature_m_×0.501+humiditym×0.152−22.037; AIC=856.708; likelihood ratio chi-square=849.017; Model 4, 3-month time lag: log D_m_=rainfall_m_×0.006+temperature_m_×0.786+humiditym×0.071−26.331; AIC=855.721; likelihood ratio chi-square=849.163; D, number of dengue cases; m, month; AIC, Akaike information criterion.

**Table 4.** Models based on a negative binomial generalized linear model relating the monthly number of dengue cases with the rainfall, temperature, relative humidity, and season

	Variables	Estimates	Standard error	Wald chi-square
Model 1	Constant	19.838	1.683	138.879
	Rainfall	0.003	0.002	2.627
	Temperature	0.382	0.029	173.870
	Humidity	0.038	0.016	5.240
	Season	4.388	0.326	181.208
Model 2	Constant	23.025	1.882	149.706
	Rainfall	0.012	0.001	56.665
	Temperature	0.314	0.032	98.812
	Humidity	0.058	0.014	16.749
	Season	4.794	0.327	214.734
Model 3	Constant	22.265	1.791	154.537
	Rainfall	0.005	0.001	11.728
	Temperature	0.475	0.042	129.371
	Humidity	0.106	0.013	61.217
	Season	1.397	0.260	28.908
Model 4	Constant	26.409	2.036	168.249
	Rainfall	0.006	0.001	30.127
	Temperature	0.843	0.055	231.656
	Humidity	0.135	0.015	77.465
	Season	2.265	0.349	42.173

Model 1, no time lag: log D_m_=rainfall_m_×0.003+temperature_m_×0.382+hu-miditym×0.038+season×4.388−19.838; AIC=1331.447; likelihood ratio chi-square=652.496; Model 2, 1-month time lag: log D_m_=rainfall_m_×0.012+temperature_m_×0.314 +humiditym×0.058+season×4.794–23.025; AIC= 1020.722; likelihood ratio chi-square=956.592; Model 3, 2-month time lag: log D_m_=rainfall_m_×0.005+temperature_m_×0.475 +humiditym×0.106+season×1.397–22.265; AIC=959.024; likelihood ratio chi-square=1,012.036; Model 4, 3-month time lag: log D_m_=rainfall_m_×0.006+temperature_m_×0.843 +humiditym×0.135–Season×2.265–26.409; AIC=932.544; likelihood ratio chi-square=1,031.670; D, number of dengue cases; m, month; AIC, Akaike information criterion. Season code, 1: February, March, April, and May (pre-monsoon season); 2: June, July, August, and September (monsoon season); 3: October, November, December, and January (post-monsoon season).

**Table 5.** Mean Breteau index (BI) before, during, and after dengue outbreaks in East Delhi (2007-2009)

Years	BI					
	Jun - Aug		Sep - Nov		Dec - Feb	
	Case	Control	Case	Control	Case	Control
2007	0.5	0.6	2.5	1.5	<0.1	< 0.1
2008	0.9	0.8	8.1	1.8	0.1	< 0.1
2009	0.3	0.5	4.3	1.9	< 0.1	0.1
